# Single-Cell RNA-Seq Reveals Heterogeneous lncRNA Expression in Xenografted Triple-Negative Breast Cancer Cells

**DOI:** 10.3390/biology10100987

**Published:** 2021-09-30

**Authors:** Holly R. Pinkney, Michael A. Black, Sarah D. Diermeier

**Affiliations:** 1 Department of Biochemistry, University of Otago, Dunedin 9016, New Zealand; holly.pinkney@postgrad.otago.ac.nz (H.R.P.); mik.black@otago.ac.nz (M.A.B.); 2 Amaroq Therapeutics Ltd., Dunedin 9016, New Zealand

**Keywords:** single cell RNA-seq, non-coding RNAs, long non-coding RNAs, scRNA-seq, lncRNAs, ncRNAs, breast cancer, triple-negative breast cancer, TNBC, xenograft, heterogeneity

## Abstract

**Simple Summary:**

Triple-negative breast cancer (TNBC) is an aggressive subtype of breast cancer and requires further research to identify new targeted treatments. We set out to study long non-coding RNAs (lncRNAs), an emerging class of oncogenes, in the context of TNBC. As lncRNAs are expressed in a highly specific manner, we applied single-cell RNA sequencing as a high resolution method to study lncRNA expression in the tumour. Our findings demonstrate that lncRNAs are expressed heterogeneously and identify previously uncharacterised lncRNAs that can be further investigated as therapeutic targets or biomarkers.

**Abstract:**

Breast cancer is the most commonly diagnosed cancer in the world, with triple-negative breast cancer (TNBC) making up 12% of these diagnoses. TNBC tumours are highly heterogeneous in both inter-tumour and intra-tumour gene expression profiles, where they form subclonal populations of varying levels of aggressiveness. These aspects make it difficult to study and treat TNBC, requiring further research into tumour heterogeneity as well as potential therapeutic targets and biomarkers. Recently, it was discovered that the majority of the transcribed genome comprises non-coding RNAs, in particular long non-coding RNAs (lncRNAs). LncRNAs are transcripts of >200 nucleotides in length that do not encode a protein. They have been characterised as regulatory molecules and their expression can be associated with a malignant phenotype. We set out to explore TNBC tumour heterogeneity in vivo at a single cell level to investigate whether lncRNA expression varies across different cells within the tumour, even if cells are coming from the same cell line, and whether lncRNA expression is sufficient to define cellular subpopulations. We applied single-cell expression profiling due to its ability to capture expression signals of lncRNAs expressed in small subpopulations of cells. Overall, we observed most lncRNAs to be expressed at low, but detectable levels in TNBC xenografts, with a median of 25 lncRNAs detected per cell. LncRNA expression alone was insufficient to define a subpopulation of cells, and lncRNAs showed highly heterogeneous expression patterns, including ubiquitous expression, subpopulation-specific expression, and a hybrid pattern of lncRNAs expressed in several, but not all subpopulations. These findings reinforce that transcriptionally defined tumour cell subpopulations can be identified in cell-line derived xenografts, and uses single-cell RNA-seq (scRNA-seq) to detect and characterise lncRNA expression across these subpopulations in xenografted tumours. Future studies will aim to investigate the spatial distribution of lncRNAs within xenografts and patient tissues, and study the potential of subclone-specific lncRNAs as new therapeutic targets and/or biomarkers.

## 1. Introduction

Breast cancer is the most commonly diagnosed cancer worldwide, with 2.26 million cases diagnosed and over 680,000 deaths in 2020 [1]. Triple negative breast cancers (TNBC) account for approximately 12% of breast cancers, and are defined as cancers that are hormone receptor negative, lacking significant expression of estrogen receptor (ER), progesterone receptor (PR) and human epidermal growth factor receptor (HER2) [2]. TNBC is highly heterogeneous in terms of both inter-tumour diversity, and intra-tumour gene expression [3], where tumours are comprised of multiple subclonal populations, with varying levels of aggressiveness [4,5]. Tumour heterogeneity makes effective treatment difficult (as reviewed in [3]). Further research into the underlying biology of TNBC is required to better understand tumour heterogeneity, leading to the identification of new therapeutic targets and biomarkers and ultimately to improve disease management and patient outcomes.

Recently, it was discovered that the majority of the transcribed genome comprises non-coding RNAs (ncRNAs) [6]. While previous exploration of gene expression profiles in TNBC had largely been directed towards protein-coding genes [7], interest in changes to ncRNAs has been growing in recent years (as reviewed in [8]). The largest class of ncRNAs are long non-coding RNAs (lncRNAs), which are defined as transcripts of >200 nucleotides in length that do not encode a protein [9]. With some well-characterised exceptions, such as *MALAT1* [10] and *NEAT1* [11], lncRNAs are generally expressed at low levels within the cell [12,13]. LncRNAs have been implicated in numerous diseases, and have been characterised as drivers in many types of cancers [14,15,16], including TNBC [17]. They have been shown to contribute to the six hallmarks of cancer, using many mechanisms to promote the malignant state [18,19]. LncRNAs are highly suited as oncology targets due to their superior tissue- and cancer-specific expression compared to protein-coding genes [20], making them an important area to investigate in the context of TNBC.

While it is known that subpopulations of tumour cells are present in TNBC and gene expression in the tumour is highly heterogeneous [3,4], investigation of lncRNA expression within and between tumours has not been investigated in detail yet. LncRNAs have been noted to be of interest in TNBC [21,22], but little is known about their expression patterns, or how they are expressed in relation to the formation of subpopulations within a tumour. Further investigation is required to determine whether expression of specific lncRNAs may contribute to certain characteristics of these cell populations, such as enhanced invasive or proliferative potential, as lncRNAs have been described as drivers of these processes [23,24].

We set out to explore tumour heterogeneity in vivo at the single cell level to investigate whether lncRNA expression varies across different cells within the same tumour even if they were grafted from the same cell line, and whether lncRNA expression can be used to define subpopulations within the tumour. Historically, biomarkers and therapeutic targets have been identified using microarrays and bulk RNA-seq. However, these methods can result in a bias towards genes that are expressed at high levels on a population average, masking the expression of genes that have high expression in specific but small subpopulations [25]. As lncRNAs are often expressed in a highly specific fashion, the use of bulk RNA-seq may result in losing the expression signal of some lncRNAs in small subsets of cells. This phenomenon has been demonstrated in previous single-cell RNA sequencing (scRNA-seq) experiments, such as in the developing human neocortex. Many lncRNAs identified were expressed abundantly in specific cell types, but overall were found to be lowly expressed at the population level [26]. In the context of cancer, scRNA-seq allows for the identification of individual cells and small subpopulations, for example, at the leading edge of the tumour, that may differ in their lncRNA expression profiles due to their invasive potential, and/or being exposed to a different microenvironment, and is therefore a superior tool for detection of cell-specific lncRNA expression [27].

Here, we show that heterogeneity exists within a cultured cell line and that tumour cell subpopulations are present when grown in vivo in xenografts. LncRNA expression was found to be heterogeneous, with some lncRNAs such as *MALAT1*, *NEAT1* and *CYTOR* expressed at high levels on a population average, while other lncRNAs including *LINC01615*, *LOC107986152*, *MANCR* and *LOC107986265* were associated with subpopulations of cells in the xenograft. Our findings reinforce that lncRNAs play an important role in TNBC, and suggest scRNA-seq allows identification of subpopulations defined by small transcriptional changes to within a tumour.

## 2. Methods and Materials

### 2.1. Sample Preparation

**Figure 1:**
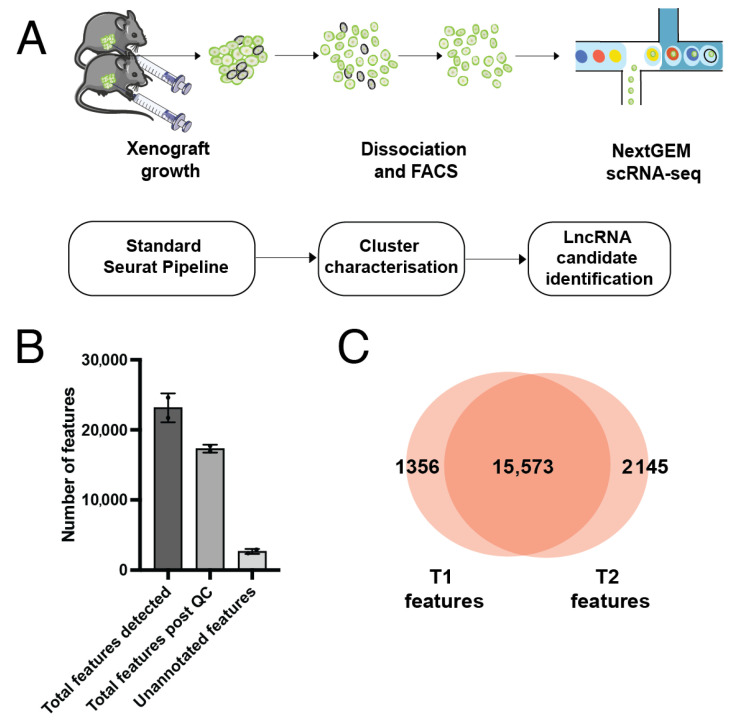
Growth and sequencing of cell-line derived xenografts. (**A**) Outline of methods used. Xenografts were grown in vivo, FACS sorted for GFP+ cells and single cell sequenced using 10× technology. Data was analysed in R using the Seurat package, and clusters were further characterised and investigated for lncRNA expression. (**B**) Feature detection pre and post QC. A low number of unannotated features were also detected. Error bars indicate the mean of two biological replicates ± stdev. (**C**) Overlap of detected features between the two samples. T1 = Tumour 1, T2 = Tumour 2.

Samples were prepared, sequenced and mapped to GrCh38 v15 as described in detail in [28]. Briefly, MDA-MB-231-LM2 cells [29] were injected into the mammary fat pad of two Nu/J mice, and tumour growth was observed for eight weeks using in vivo bioluminescence. Tumours were dissociated into single cells, and fluorescence-activated cell sorting (FACS) for GFP+ cells was performed to exclude any host mouse cells. Libraries were prepared from GFP+ cells using the 10X Chromium scRNA-seq kit, and sequenced on an Illumina platform. Libraries were mapped to GRCh38 v15 using 10x Genomics Cellranger 5.0 [30] (Figure 1A).

### 2.2. Quality Control

Following mapping, filtered feature-barcode expression values were further filtered for cell quality, normalised and scaled in R version 4.0.2 [31] using the Seurat 4.0 analysis workflow [32]. Low quality cells with abnormal feature counts per cell (high or low) or those with a high amount of mitochondrially encoded genes (suggesting the cell was undergoing apoptosis) were excluded. Specifically, cells were excluded if they had <200 or >2000 features, or >2.5% mitochondrially encoded features. Normalisation and scaling were performed using the default settings for the NormalizeData and ScaleData functions. Because the tumours were grown from a cultured epithelial cell line and further FACS sorted for specificity, cell type annotation was not required. However, to determine if any host cells had contaminated the FACS sorted samples, cell type annotation was performed using the SingleR [33] package, with the CHETAH [34] tumour microenvironment reference set.

### 2.3. Principle Component Selection and Clustering

To identify the genes most likely to define subpopulations within the tumours, the top 2000 most variable features were selected to carry forward for further analysis. The default ‘vst’ selection method in the FindVariableFeatures function was used. To reduce complexity for further analysis, a principal component analysis (PCA) was performed, and an Elbow Plot (Figure S1) was used to determine the number of components to include in downstream analyses. To cluster the data, the FindNeighbours and FindClusters functions were used on the first 15 principle components for each dataset. Granularity was not restricted. Clustering of the first two principal components for each dataset was visualised using the Uniform Manifold Approximation and Projection (UMAP) and t-distributed Stochastic Neighbor Embedding (t-SNE) methods. For both samples, the top 10 most highly expressed genes per cluster were visualised on a heatmap.

### 2.4. Cluster Characterisation and lncRNA Identification

To determine which genes were upregulated and define a given cluster, differential expression analysis between a given cluster compared to all other cells was performed using the FindAllMarkers function. The following parameters were used: to be significantly upregulated and defining a cluster, a gene must be found in at least 25% of the cluster, have a minimum log2 fold change (log2FC) of +0.25, and have an adjusted *p*-value of *p*≤ 0.05 (Bonferroni adjustment). Significant upregulation in one cluster did not exclude lower levels of expression in other clusters.

Clusters were investigated to determine similarity between samples. To gain an approximation of whether subpopulations are functionally distinct, gene function patterns were investigated for significantly upregulated genes in clusters of both samples using Metascape [35]. Upregulated genes were then filtered for lncRNAs using the GENCODE GRCh38.p13 lncRNA annotation file [36], with manual investigation of unannotated loci. LncRNA expression across clusters was investigated using the FeaturePlot function.

To validate our findings, we identified upregulated lncRNAs and investigated the expression patterns in an additional scRNA-seq dataset of MDA-MB-231 cells which were grown in vitro (compared to the xenografts in our study which were grown in vivo (accession number GSE181410). MDA-MB-231 cells are the parental cell line to the MDA-MB-231-LM2 cell line used in this study, and while not identical, are a relatively comparable control. This data set was downloaded as an h5 Seurat object, with quality control and clustering already performed by the authors. We performed marker identification and lncRNA filtering and analysis to this data set as described for our xenografted cells described above.

## 3. Results

### 3.1. Quality Control

**Figure 2:**
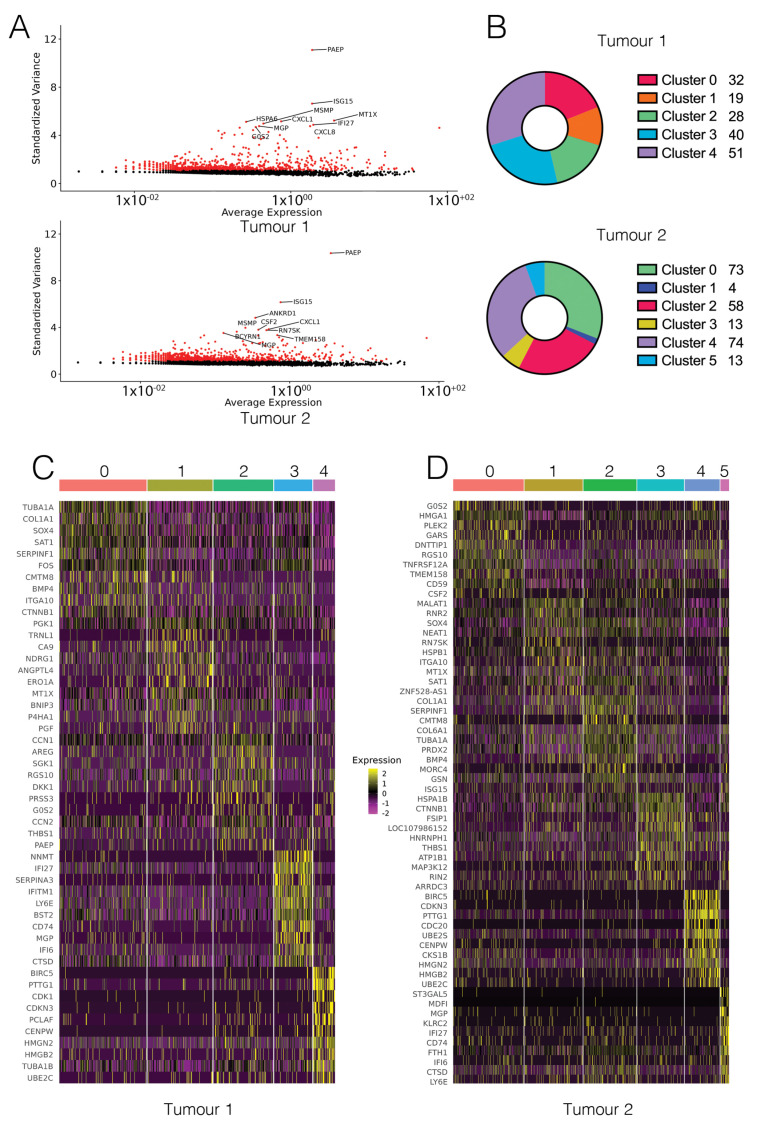
Subpopulations in cell-line derived xenografts form clusters. (**A**) The top 2000 most variable genes for each sample were highly comparable, and included genes known to be associated with cancer phenotypes. (**B**) Number of significantly upregulated genes for each cluster. Clusters are coloured by similarity between T1 and T2. (**C**) Heatmap showing the top 10 genes for each T1 cluster, coloured by expression (Log2FC). (**D**) Heatmap showing the top 10 genes for each T2 cluster, coloured by expression (Log2FC).

We detected expression from a total of 21,701 genes from 695 cells in Tumour 1 (T1), and 24,606 genes from 799 cells in Tumour 2 (T2) (Figure S2). The median gene (feature) count per cell was 1802 for T1 and 1919 for T2 (Figure S3). Following filtering for low quality cells (based on feature counts per cell and percentage of mitochondrial genes, Figure S4), there were 16,929 features across 514 cells remaining for T1 and 17,718 features across 687 cells remaining for T2 (Figure 1B). Of these, 15,573 were common between both T1 and T2 (Figure 1C). A small number of features were detected that were unable to be matched with data in the GENCODE reference file and were therefore classed as ‘unannotated’. Selection of the top 2000 most variable features excluded all genes with a standardised variance of approximately <1.5 (Figure 2A). The most highly variable features in both samples were protein-coding genes known to be associated with cancer. These include genes such as *PAEP*, which encodes the protein glycodelin, a lipocalin protein associated with reproductive cancers including breast cancer [37], *ISG15*, a prognostic marker in breast cancer [38], and *ANKRD1*, which has been associated with lung and ovarian cancer [39,40] (Figure 2A). Cell type annotation did not result in meaningful calls. To confirm this, reference cell type expression was correlated with expression centroids for each cell in T1 and T2. Both T1 and T2 cells had a median Pearson correlation coefficient of 0.37 between cell expression centroids and the reference cell expression, with no correlation with a reference cell type being greater than 0.5 (Figure S7). This confirmed cell type calls were not based on a strong correlation to the reference cell type, indicating that the dataset was likely not contaminated with mouse host cells, and instead represents a predominantly epithelial cell population.

### 3.2. Cluster Characterisation

**Figure 3:**
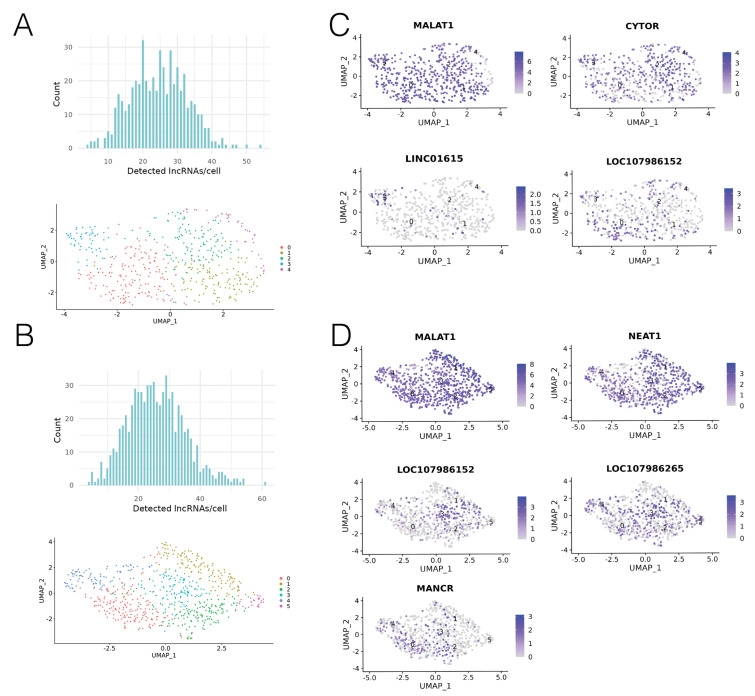
LncRNA expression was heterogeneous in cell line derived xenografts. (**A**) Number of T1 lncRNAs expressed per cell (count = number of cells), UMAP projection of cell clustering, coloured by cluster. (**B**) Number of T2 lncRNAs expressed per cell (count = number of cells), UMAP projection of cell clustering, coloured by cluster. (**C**) T1 upregulated lncRNA expression across cells. Scale = Log2FC. (**D**) T2 upregulated lncRNA expression across cells. Scale = Log2FC.

Clustering using FindNeighbours and FindClusters resulted in five clusters for T1 and six clusters for T2 (Figure 2B). The FindAllMarkers function was used to perform differential gene expression analysis between a given cluster compared to all other cells. Cluster visualisation on a UMAP projection showed two major clusters for T1 and three major clusters for T2, with the remaining clusters in each sample having a smaller number of significantly upregulated genes associated with them (Figure 2B and Figure 3A,B). For T2, cluster 1 had only four significantly upregulated genes. In total, there were 429 significantly upregulated genes in common between T1 and T2. Genes and pathways previously identified as dysregulated in TNBC were detected in our dataset. These include the upregulation of genes involved in MAPK [41] and NFkB [42] signalling, and cyclin dependent kinases [43].

Visualising the top 10 genes for each cluster using a heatmap revealed that for both T1 and T2, the top 10 genes defined each cluster in a specific manner (Figure 2C,D), with structure evident across the heatmap. Clusters were also visualised on a t-SNE projection (Figure S5). Common clusters were identified between T1 and T2, with four out of five T1 clusters matching clusters in T2 (Table 1 and S1–S5). While analysis was limited by the small number of genes defining each cluster (Figure 2B), gene function analysis using Metascape suggested that the common subpopulations in T1 and T2 were clustering due to changes in expression of small number of genes including some genes involved in extracellular matrix remodelling and cell signalling (Figure S6).

### 3.3. Significantly Upregulated lncRNAs

In general, expression levels of most lncRNAs across T1 and T2 were low, indicating that scRNA-seq was beneficial for detection of lowly expressed transcripts. From the total 16,929 features for T1 and 17,718 features for T2, 1069 annotated lncRNAs were detected in T1, and 1154 annotated lncRNAs were detected in T2. From the 2000 most variable genes in T1 and T2, the FindAllMarkers function identified seven lncRNAs which were significantly upregulated in one of the clusters, compared to all other cells (Table 2, Figure 3C,D). The low number of upregulated lncRNAs indicates that most of the detected lncRNAs were expressed at a similar level across the entire xenografted tumour, and were not inherent to any subpopulation. We found that lncRNAs were present in all clusters, with a median of 25 lncRNAs detected per cell (Figure 3A,B). We then classified the expression profiles of the seven upregulated lncRNAs. LncRNAs that were significantly upregulated in one cluster with low/no levels of expression in all other clusters were classed as having “cluster-specific” expression. LncRNAs that were significantly upregulated in one cluster, but were also present in all other clusters were classed as having “ubiquitous” expression. Lastly, lncRNAs that were not cluster-specific or ubiquitously expressed (i.e., were expressed in more than one but not all clusters) were classed as having a “hybrid” expression profile.

Analysis of T1 revealed four lncRNAs which exhibited significantly higher expression in one cluster compared to all other cells. These were *MALAT1*, *CYTOR*, *LINC01615* and *LOC107986152* (Figure 3A). As *MALAT1* was significantly upregulated in cluster 0, but was also expressed at a lower level across almost all cells in the sample, it was classed as having ubiquitous expression. In a similar fashion, *CYTOR* was significantly upregulated in cluster 2, however, it was also expressed at a lower level in all other clusters and therefore also classed as having ubiquitous expression. *LINC01615* was found to be significantly upregulated in cluster 3, and was expressed in almost no cells outside of cluster 3, leading us to classify expression of *LINC01615* as cluster-specific. *LOC107986152* was found to be significantly upregulated in cluster 0. It also showed some lower expression in other clusters, which led us to classify it as having a hybrid expression pattern.

Analysis of T2 revealed five significantly upregulated lncRNAs. These were *MALAT1*, *LOC107986152*, *LOC107986265*, *MANCR* and *NEAT1* (Figure 3C). *MALAT1* was significantly upregulated in cluster 1 compared to all other cells. Similarly to its expression pattern in T1, *MALAT1* was also expressed at a lower level in almost every cell in the sample. *LOC107986152* was significantly upregulated in cluster 3, and similar to its expression pattern in T1, had some lower expression in other clusters. *LOC107986265* was upregulated in cluster 3, but was present at lower levels in all clusters, and so was classed as having a ubiquitous expression pattern. *MANCR* was significantly upregulated in cluster 0, with some lower expression in other clusters, and was therefore classed as having a hybrid expression pattern. Lastly, *NEAT1* was significantly upregulated in cluster 1, and also showed a lower level of expression across almost all cells in the sample, resulting in its classification as ubiquitously expressed.

To validate our findings, we also analysed a publically available scRNA-seq dataset from MDA-MB-231 cells. The dataset contained data for 38,702 features over 4374 cells, and was sequenced more deeply than T1 and T2 (Figure S8A), however, we detected a comparable number of lncRNAs per cell (Figure S8B), confirming that our dataset was sequenced to sufficient depth for lncRNA detection. Additionally, we identified overlaps in the list of significantly upregulated lncRNAs, with *MALAT1*, *NEAT1* and *MANCR* upregulated, and following very similar expression patterns to those found in xenografts (Figure S8C–E). We also observed *CYTOR* and *LINC01615* to be expressed in the MDA-MB-231 dataset but not significantly upregulated in any cluster.

## 4. Discussion

We used scRNA-seq to investigate the heterogeneity of lncRNA expression in vivo using TNBC xenografts. The resulting tumours were found to cluster into cellular subpopulations. While some cell to cell variation in expression is expected in cell lines [44], transcriptional subpopulations of cells may also (at least, in part) have evolved over the growth period of 8 weeks in the mouse mammary fat pad. The identified subpopulations clustered based on changes in the expression of small numbers of genes (<100 of 17,000 genes detected in total), and could potentially represent different spatial locations within the tumour, where oxygen and metabolite gradients, as well as interactions with the tumour microenvironment can affect tumour growth [45,46]. The major clusters found in T1 corresponded well with clusters in T2 based on gene expression profiles. While pathway analysis was based on a relatively small number of genes, clustering appeared to correlate with changes to biological processes such as extracellular remodelling, which is often leveraged in cancer [47], and upregulation of components involved in cell signalling, which can play a number of roles in cancer [48]. LncRNA expression alone was insufficient to define a cluster, however, clusters were able to be stratified by their top 10 genes.

Because lncRNAs are regulatory molecules that can drive the malignant phenotype [19], we focussed our investigation on changes in lncRNA expression across the two samples to understand whether they played a role in the formation of subpopulations within the tumour. We identified seven significantly upregulated lncRNAs, but found that their patterns of expression were heterogeneous. *MALAT1*, *NEAT1* and *CYTOR* had a ubiquitous expression pattern, with high levels of expression on population average. Because of their ubiquitous expression, it is unlikely that these lncRNAs are drivers behind the formation of subpopulations in the tumour. *MALAT1* was expressed more highly than any other lncRNA in both T1 and T2, which is in agreement with the literature [10]. *MALAT1* has been shown to be a prognostic marker in TNBC [49], and implicated in therapeutic resistance [50] and metastasis in breast cancer [51]. It appears to affect the malignant phenotype by chromatin binding, and as a splicing modulator [52]. *NEAT1* has been shown to be involved in many cancers [53,54], and promotes chemoresistance in TNBC [17], while early studies on the lncRNA *CYTOR* (also known as *LINC00152*) have shown it may regulate signalling in breast cancers [55,56].

*LINC01615* was expressed at lower levels on a population average and was classed as having a cluster-specific expression pattern. Initial studies on *LINC01615* demonstrate that it may be involved in metastasis in hepatocellular carcinomas [57,58]. As MDA-MB-231-LM2 cells are highly metastatic [29], the expression of *LINC01615* may suggest that it is also associated with metastasis in TNBC. The cluster-specific expression of this lncRNA, implies that perhaps that particular subpopulation may have a higher tendency to metastasise than the other populations in the tumour.

*LOC107986152*, *LOC107986265* and *MANCR* had an expression pattern that was a hybrid of the two classes, being significantly upregulated in one cluster, but with moderate expression in additional clusters. *LOC107986152* was found to be significantly upregulated in both T1 and T2, and is thus far an uncharacterised lncRNA. Its genomic size is 12,782 bp, and it comprises two exons forming a transcript of 3402 nucleotides in length. *LOC107986152* is located closely upstream and antisense to *GOLIM4* on chromosome 3, a gene that encodes a golgi integral membrane protein [59]. *LOC107986265* is another thus far uncharacterised lncRNA. Its genomic size is 653 bp and it comprises two exons forming a transcript of 591 nucleotides in length. It completely overlaps with the gene *GBA3* in the sense direction on chromosome 4. *GBA3* is a gene which encodes a cytosolic beta-glucosidase [60]. Further characterisation of these two novel lncRNAs could be performed using our previously described lncRNA toolkit [61]. Finally, early studies suggest depletion of the lncRNA *MANCR* has been shown to reduce cell proliferation and viability in TNBC [62].

We demonstrate that scRNA-seq can be used to identify potentially oncogenic lncRNAs within aggressive subpopulations of patient tumours. Additionally, the previously uncharacterised lncRNAs found to be upregulated in a cluster-specific manner have the potential to be further characterised in the context of TNBC progression, which may lead to the identification of novel biomarkers and/or therapeutic targets in the future.

The conclusions from this study are limited as only two xenografted tumours were analysed, making generalisation of conclusions across TNBC xenografts difficult. While the overlap in overall gene expression between the two samples was very high, the overlap between significantly upregulated genes was limited, with some clusters being unable to be matched between the two tumour samples. This could be attributed to heterogeneity within the cell line prior to xenograft growth, or inter-individual variation of transcriptional patterns in vivo. The analysis of additional TNBC xenografted tumour samples would address these limitations and increase the resolution of gene expression in subpopulations. The analysis of an independent scRNA-seq dataset of MDA-MB-231 cells revealed an overlap in lncRNA expression with our dataset, and these lncRNAs followed similar expression patterns, adding further validity to our dataset and analysis. The detection of *MALAT1*, *NEAT1* and *MANCR* in MDA-MB-231 cells grown in vitro suggests that these lncRNAs may always be upregulated in the MDA-MD-231 model of TNBC, while the detection but not significant upregulation of *CYTOR* and *LINC01615* suggests that there may be differences in lncRNA expression between in vitro and in vivo growth. Alternatively, these lncRNAs may differ in their expression patterns between MDA-MB-231 and MDA-MB-231-LM2 cells.

Future experiments will aim to determine the potential of any of the seven identified upregulated lncRNAs as oncology targets, and to understand their expression in the spatial context of the tumour. Tumour cell subpopulations often form according to their spatial positioning within the tumour, and some subpopulations may have an increased ability to metastasise. For example, the spatial expression of *LINC01615* would be important to consider, as it has the potential to be associated with metastasis in TNBC. To determine spatial expression patterns, RNA in situ hybridisation could be performed on formalin fixed, paraffin embedded (FFPE) sections of TNBC xenografted tumours. LncRNAs expressed at the invasive edge of the tumour could be further explored in patient-derived TNBC tissue samples, and could be followed up by a more detailed investigation of lncRNA expression in circulating tumour cells and metastatic nodules.

## 5. Conclusions

In summary, the present study addresses the underlying biology of TNBC, with the overarching goal of finding potential oncology targets to improve patient-specific treatment and monitoring of disease. LncRNAs are an increasingly important group of oncology targets, which have been shown to act as drivers in TNBC, and are also emerging as clinical biomarkers [19,63]. We have shown that lncRNAs are overall expressed at low levels in TNBC xenografted cells, but are detectable using scRNA-seq. We showed that lncRNAs are expressed in a heterogeneous fashion that can be classed as ubiquitous, cluster-specific, or hybrid. We also identified lncRNAs that have not been characterised in detail in TNBC, including two novel lncRNAs, *LOC107986152* and *LOC107986265*, and should be further investigated as oncology targets.

## Figures and Tables

**Table 1:** Matching clusters between T1 and T2.

T1 Cluster	T2 Cluster	% Markers in Assigned Cluster
0	2	72%
1	Undetermined	-
2	0	71%
3	5	67%
4	4	92%
Undetermined	1	-
Undetermined	3	-

**Table 2:** Significant upregulated lncRNAs.

LncRNA	Identified in:	Log2FC	Adjusted *p*-Value	Cluster
*MALAT1*	T1, T2	1.1, 1.4	9.75 × 10^−04^, 1.60 × 10^−16^	0, 1
*LOC107986152*	T1, T2	0.76, 1.2	3.22 × 10^−02^, 1.02 × 10^−11^	0, 3
*LINC01615*	T1	1.07	1.12 × 10^−07^	3
*CYTOR*	T1	0.76	2.51 × 10^−04^	2
*LOC107986265*	T2	0.93	1.33 × 10^−04^	3
*MANCR*	T2	0.32	1.91 × 10^−03^	0
*NEAT1*	T2	0.70	3.54 × 10^−03^	1

## Data Availability

All data is available in the NCBI GEO repository under the accession number GSE163210. Findings were validated using data from NCBI GEO under the accession number GSE181410.

## Supplementary Materials

The following are available online at www.mdpi.com/xxx/, Figure S1: Elbow plots of principle component variation, Figure S2: Sequencing summaries, Figure S3: Features per cell information, Figure S4: Cell distributions prior to QC measures, Figure S5: Clustering visualised on a t-SNE projection, Figure S6: Metascape analysis for GO terms and pathways associated with common clusters between T1 and T2, Figure S7: Cell type annotation QC, Figure S8: extra dataset analysis. Table S1: Common clustering in T1 and T2, Table S2: Common genes between T1 cluster 0 and T2 cluster 2, Table S3: Common genes between T1 cluster 2 and T2 cluster 0, Table S4: Common genes between T1 cluster 3 and T2 cluster 5, Table S5: Common genes between T1 cluster 4 and T2 cluster 4.

## Abbreviations

The following abbreviations are used in this manuscript:

RNA	Ribonucleic acid
RNA-seq	RNA sequencing
stdev	Standard Deviation

## References

[1] Ferlay J., Ervik M., Lam F., Colombet M., Mery L., Piñeros M., Znaor A., Soerjomataram I., Bray F. (2020). Global Cancer Observatory: Cancer Today.

[2] Alteri R., Barnes C., Fedewa S., Gansler T., Gaudet M.M., Gierach G., Kalidas M., Kramer J., McMahon K., Miller K. (2019). Breast Cancer Facts and Figures 2019–2020. https://www.cancer.org/content/dam/cancer-org/research/cancer-facts-and-statistics/breast-cancer-facts-and-figures/breast-cancer-facts-and-figures-2019-2020.pdf.

[3] Koren S., Bentires-Alj M. (2015). Breast Tumor Heterogeneity: Source of Fitness, Hurdle for Therapy. Mol. Cell.

[4] Shah S.P., Roth A., Goya R., Oloumi A., Ha G., Zhao Y., Turashvili G., Ding J., Tse K., Haffari G. (2012). The clonal and mutational evolution spectrum of primary triple-negative breast cancers. Nature.

[5] Karaayvaz M., Cristea S., Gillespie S.M., Patel A.P., Mylvaganam R., Luo C.C., Specht M.C., Bernstein B.E., Michor F., Ellisen L.W. (2018). Unravelling subclonal heterogeneity and aggressive disease states in TNBC through single-cell RNA-seq. Nat. Commun..

[6] The ENCODE Project Consortium (2012). An integrated encyclopedia of DNA elements in the human genome. Nature.

[7] Criscitiello C., Azim H.A., Schouten P.C., Linn S.C., Sotiriou C. (2012). Understanding the biology of triple-negative breast cancer. Ann. Oncol..

[8] Liu J., Zhao G., Liu X.L., Zhang G., Zhao S.Q., Zhang S.L., Luo L.H., Yin D.C., Zhang C.Y. (2021). Progress of non-coding RNAs in triple-negative breast cancer. Life Sci..

[9] Kornienko A.E., Guenzl P.M., Barlow D.P., Pauler F.M. (2013). Gene regulation by the act of long non-coding RNA transcription. BMC Biology.

[10] GTEx Consortium (2017). Genetic effects on gene expression across human tissues. Nature.

[11] Hutchinson J.N., Ensminger A.W., Clemson C.M., Lynch C.R., Lawrence J.B., Chess A. (2007). A screen for nuclear transcripts identifies two linked noncoding RNAs associated with SC35 splicing domains. BMC Genom..

[12] Cabili M.N., Trapnell C., Goff L., Koziol M., Tazon-Vega B., Regev A., Rinn J.L. (2011). Integrative annotation of human large intergenic noncoding RNAs reveals global properties and specific subclasses. Genes Dev..

[13] Cabili M.N., Dunagin M.C., McClanahan P.D., Biaesch A., Padovan-Merhar O., Regev A., Rinn J.L., Raj A. (2015). Localization and abundance analysis of human lncRNAs at single-cell and single-molecule resolution. Genome Biol..

[14] Rinn J.L., Kertesz M., Wang J.K., Squazzo S.L., Xu X., Brugmann S.A., Goodnough L.H., Helms J.A., Farnham P.J., Segal E. (2007). Functional demarcation of active and silent chromatin domains in human HOX loci by noncoding RNAs. Cell.

[15] Sun M., Gadad S.S., Kim D.S., Kraus W.L. (2015). Discovery, Annotation, and Functional Analysis of Long Noncoding RNAs Controlling Cell-Cycle Gene Expression and Proliferation in Breast Cancer Cells. Mol. Cell.

[16] Diermeier S.D., Chang K.C., Freier S.M., Song J., El Demerdash O., Krasnitz A., Rigo F., Bennett C.F., Spector D.L. (2016). Mammary Tumor-Associated RNAs Impact Tumor Cell Proliferation, Invasion, and Migration. Cell Rep..

[17] Shin V.Y., Chen J., Cheuk I.W.Y., Siu M.T., Ho C.W., Wang X., Jin H., Kwong A. (2019). Long non-coding RNA NEAT1 confers oncogenic role in triple-negative breast cancer through modulating chemoresistance and cancer stemness. Cell Death Dis..

[18] Hanahan D., Weinberg R.A. (2000). The Hallmarks of Cancer. Cell.

[19] Schmitt A.M., Chang H.Y. (2016). Long Noncoding RNAs in Cancer Pathways. Cancer Cell.

[20] Ulitsky I., Bartel D.P. (2013). lincRNAs: Genomics, Evolution, and Mechanisms. Cell.

[21] Lv M., Xu P., Wu Y., Huang L., Li W., Lv S., Wu X., Zeng X., Shen R., Jia X. (2016). lncRNAs as new biomarkers to differentiate triple negative breast cancer from non-triple negative breast cancer. Oncotarget.

[22] Zhao Z., Guo Y., Liu Y., Sun L., Chen B., Wang C., Chen T., Wang Y., Li Y., Dong Q. (2021). Individualized lncRNA differential expression profile reveals heterogeneity of breast cancer. Oncogene.

[23] Liu S.J., John Liu S., Dang H.X., Lim D.A., Feng F.Y., Maher C.A. (2021). Long noncoding RNAs in cancer metastasis. Nat. Rev. Cancer.

[24] Li Y., Egranov S.D., Yang L., Lin C. (2019). Molecular mechanisms of long noncoding RNAs-mediated cancer metastasis. Genes Chromosom. Cancer.

[25] Saliba A.E., Westermann A.J., Gorski S.A., Vogel J. (2014). Single-cell RNA-seq: Advances and future challenges. Nucleic Acids Res..

[26] Liu S.J., Nowakowski T.J., Pollen A.A., Lui J.H., Horlbeck M.A., Attenello F.J., He D., Weissman J.S., Kriegstein A.R., Diaz A.A. (2016). Single-cell analysis of long non-coding RNAs in the developing human neocortex. Genome Biol..

[27] Nguyen A., Khoo W.H., Moran I., Croucher P.I., Phan T.G. (2018). Single Cell RNA Sequencing of Rare Immune Cell Populations. Front. Immunol..

[28] Moravec J.C., Lanfear R., Spector D., Diermeier S., Gavryushkin A. (2021). Cancer phylogenetics using single-cell RNA-seq data. bioRxiv.

[29] Minn A.J., Gupta G.P., Siegel P.M., Bos P.D., Shu W., Giri D.D., Viale A., Olshen A.B., Gerald W.L., Massagué J. (2005). Genes that mediate breast cancer metastasis to lung. Nature.

[30] Zheng G.X.Y., Terry J.M., Belgrader P., Ryvkin P., Bent Z.W., Wilson R., Ziraldo S.B., Wheeler T.D., McDermott G.P., Zhu J. (2017). Massively parallel digital transcriptional profiling of single cells. Nat. Commun..

[31] R Core Team (2021). R: A Language and Environment for Statistical Computing.

[32] Hao Y., Hao S., Andersen-Nissen E., Mauck W.M., Zheng S., Butler A., Lee M.J., Wilk A.J., Darby C., Zager M. (2021). Integrated analysis of multimodal single-cell data. Cell.

[33] Aran D., Looney A.P., Liu L., Wu E., Fong V., Hsu A., Chak S., Naikawadi R.P., Wolters P.J., Abate A.R. (2019). Reference-based analysis of lung single-cell sequencing reveals a transitional profibrotic macrophage. Nat. Immunol..

[34] de Kanter J.K., Lijnzaad P., Candelli T., Margaritis T., Holstege F.C.P. (2019). CHETAH: A selective, hierarchical cell type identification method for single-cell RNA sequencing. Nucleic Acids Res..

[35] Zhou Y., Zhou B., Pache L., Chang M., Khodabakhshi A.H., Tanaseichuk O., Benner C., Chanda S.K. (2019). Metascape provides a biologist-oriented resource for the analysis of systems-level datasets. Nat. Commun..

[36] Frankish A., Diekhans M., Ferreira A.M., Johnson R., Jungreis I., Loveland J., Mudge J.M., Sisu C., Wright J., Armstrong J. (2019). GENCODE reference annotation for the human and mouse genomes. Nucleic Acids Res..

[37] Cui J., Liu Y., Wang X. (2017). The Roles of Glycodelin in Cancer Development and Progression. Front. Immunol..

[38] Kariri Y.A., Alsaleem M., Joseph C., Alsaeed S., Aljohani A., Shiino S., Mohammed O.J., Toss M.S., Green A.R., Rakha E.A. (2021). The prognostic significance of interferon-stimulated gene 15 (ISG15) in invasive breast cancer. Breast Cancer Res. Treat..

[39] Takahashi A., Seike M., Chiba M., Takahashi S., Nakamichi S., Matsumoto M., Takeuchi S., Minegishi Y., Noro R., Kunugi S. (2018). Ankyrin Repeat Domain 1 Overexpression is Associated with Common Resistance to Afatinib and Osimertinib in EGFR-mutant Lung Cancer. Sci. Rep..

[40] Lei Y., Henderson B.R., Emmanuel C., Harnett P.R., de Fazio A. (2015). Inhibition of ANKRD1 sensitizes human ovarian cancer cells to endoplasmic reticulum stress-induced apoptosis. Oncogene.

[41] Loi S., Dushyanthen S., Beavis P.A., Salgado R., Denkert C., Savas P., Combs S., Rimm D.L., Giltnane J.M., Estrada M.V. (2016). RAS/MAPK Activation Is Associated with Reduced Tumor-Infiltrating Lymphocytes in Triple-Negative Breast Cancer: Therapeutic Cooperation Between MEK and PD-1/PD-L1 Immune Checkpoint Inhibitors. Clin. Cancer Res..

[42] Kim J.-Y., Jung H.H., Ahn S., Bae S., Lee S.K., Kim S.W., Lee J.E., Nam S.J., Ahn J.S., Im Y.-H. (2016). The relationship between nuclear factor (NF)-*κ*B family gene expression and prognosis in triple-negative breast cancer (TNBC) patients receiving adjuvant doxorubicin treatment. Sci. Rep..

[43] Ding L., Cao J., Lin W., Chen H., Xiong X., Ao H., Yu M., Lin J., Cui Q. (2020). The Roles of Cyclin-Dependent Kinases in Cell-Cycle Progression and Therapeutic Strategies in Human Breast Cancer. Int. J. Mol. Sci..

[44] Ross D.T., Scherf U., Eisen M.B., Perou C.M., Rees C., Spellman P., Iyer V., Jeffrey S.S., Van de Rijn M., Waltham M. (2000). Systematic variation in gene expression patterns in human cancer cell lines. Nat. Genet..

[45] Carmona-Fontaine C., Deforet M., Akkari L., Thompson C.B., Joyce J.A., Xavier J.B. (2017). Metabolic origins of spatial organization in the tumor microenvironment. Proc. Natl. Acad. Sci. USA.

[46] Hu M., Polyak K. (2008). Molecular characterisation of the tumour microenvironment in breast cancer. Eur. J. Cancer.

[47] Winkler J., Abisoye-Ogunniyan A., Metcalf K.J., Werb Z. (2020). Concepts of extracellular matrix remodelling in tumour progression and metastasis. Nat. Commun..

[48] Sever R., Brugge J.S. (2015). Signal transduction in cancer. Cold Spring Harb. Perspect. Med..

[49] Ou X., Gao G., Bazhabayi M., Zhang K., Liu F., Xiao X. (2019). MALAT1 and BACH1 are prognostic biomarkers for triple-negative breast cancer. J. Cancer Res. Ther..

[50] Shaath H., Vishnubalaji R., Elango R., Khattak S., Alajez N.M. (2021). Single-cell long noncoding RNA (lncRNA) transcriptome implicates MALAT1 in triple-negative breast cancer (TNBC) resistance to neoadjuvant chemotherapy. Cell Death Discov..

[51] Arun G., Diermeier S., Akerman M., Chang K.C., Wilkinson J.E., Hearn S., Kim Y., MacLeod A.R., Krainer A.R., Norton L. (2016). Differentiation of mammary tumors and reduction in metastasis upon Malat1 lncRNA loss. Genes Dev..

[52] Arun G., Spector D.L. (2019). MALAT1 long non-coding RNA and breast cancer. RNA Biol..

[53] Hu X., Bao J., Wang Z., Zhang Z., Gu P., Tao F., Cui D., Jiang W. (2016). The plasma lncRNA acting as fingerprint in non-small-cell lung cancer. Tumour Biol..

[54] Li Y., Li Y., Chen W., He F., Tan Z., Zheng J., Wang W., Zhao Q., Li J. (2015). NEAT expression is associated with tumor recurrence and unfavorable prognosis in colorectal cancer. Oncotarget.

[55] Shen X., Zhong J., Yu P., Zhao Q., Huang T. (2019). YY1-regulated LINC00152 promotes triple negative breast cancer progression by affecting on stability of PTEN protein. Biochem. Biophys. Res. Commun..

[56] Van Grembergen O., Bizet M., de Bony E.J., Calonne E., Putmans P., Brohée S., Olsen C., Guo M., Bontempi G., Sotiriou C. (2016). Portraying breast cancers with long noncoding RNAs. Sci. Adv..

[57] Xiao Y., Hu F., Li M., Mo L., Xu C., Wang X., Nie J., Yang L., Xie B. (2020). Interaction between linc01615 and miR-491-5p regulates the survival and metastasis of colorectal cancer cells. Transl. Cancer Res..

[58] Ji D., Chen G.F., Liu X., Zhu J., Sun J.Y., Zhang X.Y., Lu X.J. (2019). Identification of LINC01615 as potential metastasis-related long noncoding RNA in hepatocellular carcinoma. J. Cell. Physiol..

[59] Natarajan R., Linstedt A.D. (2004). A cycling cis-Golgi protein mediates endosome-to-Golgi traffic. Mol. Biol. Cell.

[60] Yahata K., Mori K., Arai H., Koide S., Ogawa Y., Mukoyama M., Sugawara A., Ozaki S., Tanaka I., Nabeshima Y.I. (2000). Molecular cloning and expression of a novel klotho-related protein. J. Mol. Med..

[61] Pinkney H.R., Wright B.M., Diermeier S.D. (2020). The lncRNA Toolkit: Databases and In Silico Tools for lncRNA Analysis. Noncoding RNA.

[62] Tracy K.M., Tye C.E., Ghule P.N., Malaby H.L.H., Stumpff J., Stein J.L., Stein G.S., Lian J.B. (2018). Mitotically-Associated lncRNA (MANCR) Affects Genomic Stability and Cell Division in Aggressive Breast Cancer. Mol. Cancer Res..

[63] Loeb S., Partin A.W. (2011). Review of the literature: PCA3 for prostate cancer risk assessment and prognostication. Rev. Urol..

