# Supplementary figures and images for: Single-Cell RNA-Seq Reveals Heterogeneous lncRNA Expression in Xenografted Triple-Negative Breast Cancer Cells
Source: Biology (Basel). 2021 Sep 30;10(10):987. doi: 10.3390/biology10100987 (PMC8533545; doi:10.3390/biology10100987)

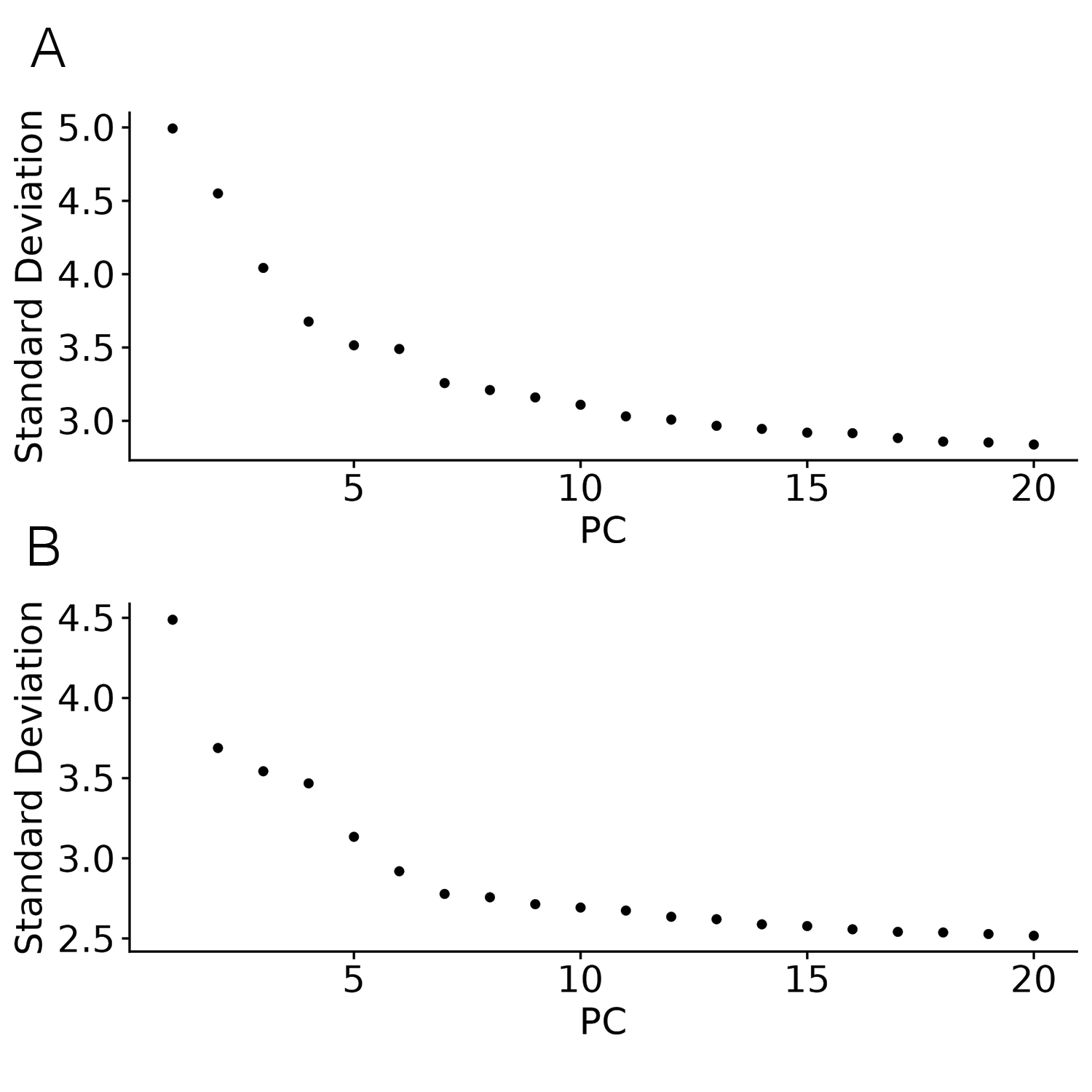

Supplement: Supplementary file 1 [file biology-10-00987-s001.zip › Supplementary_material/supl-fig-1.png]

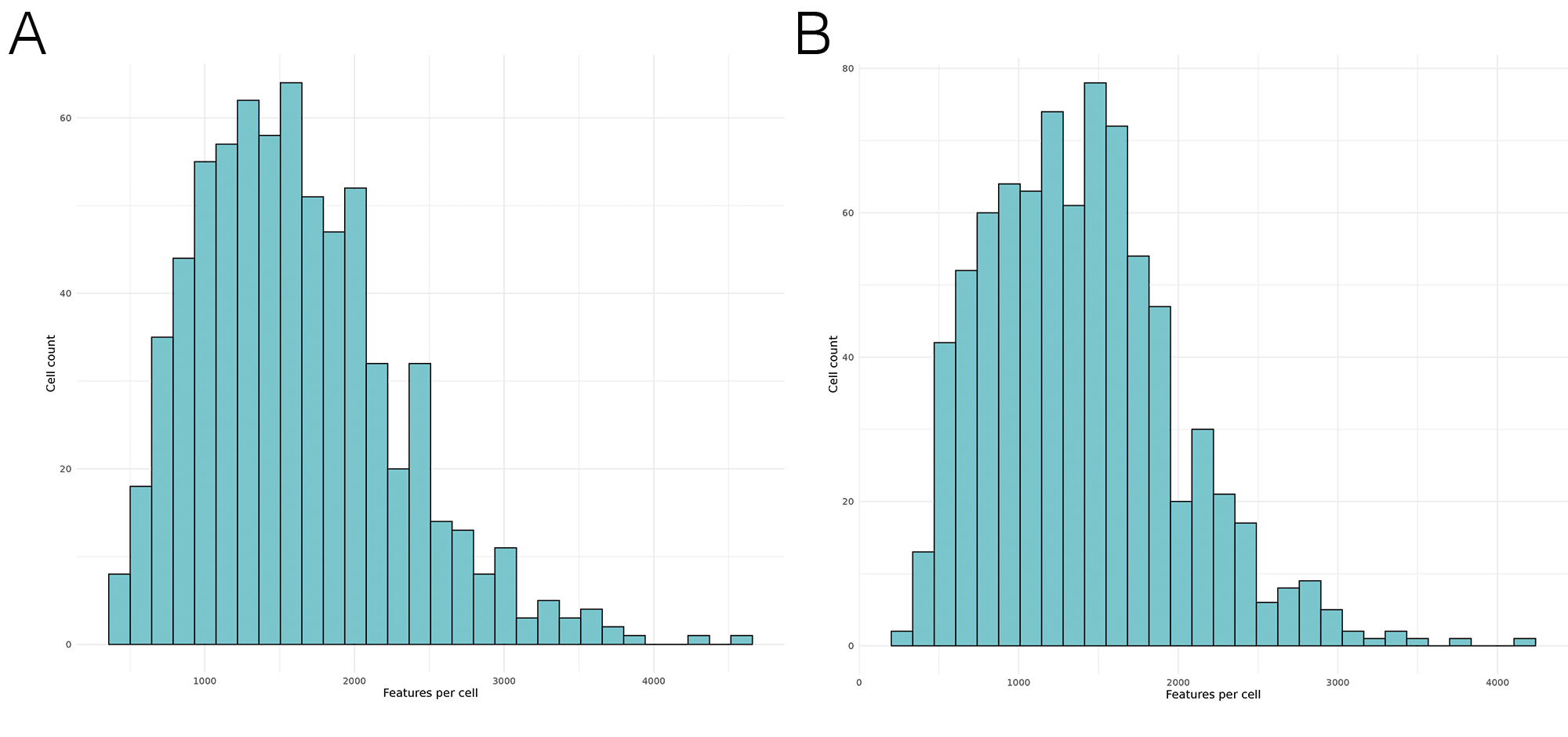

Supplement: Supplementary file 1 [file biology-10-00987-s001.zip › Supplementary_material/supl-fig-3.png]

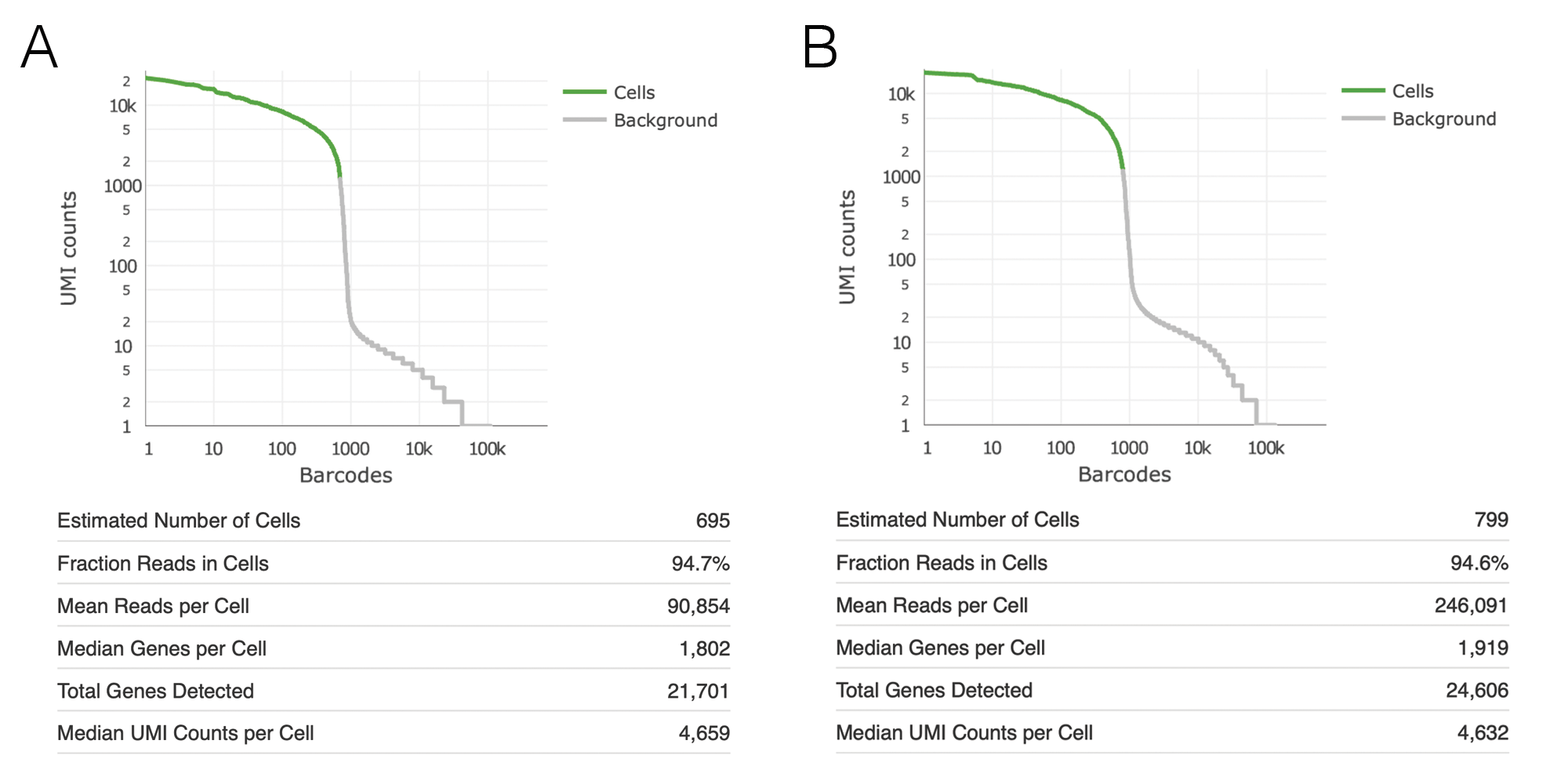

Supplement: Supplementary file 1 [file biology-10-00987-s001.zip › Supplementary_material/supl-fig-2.png]

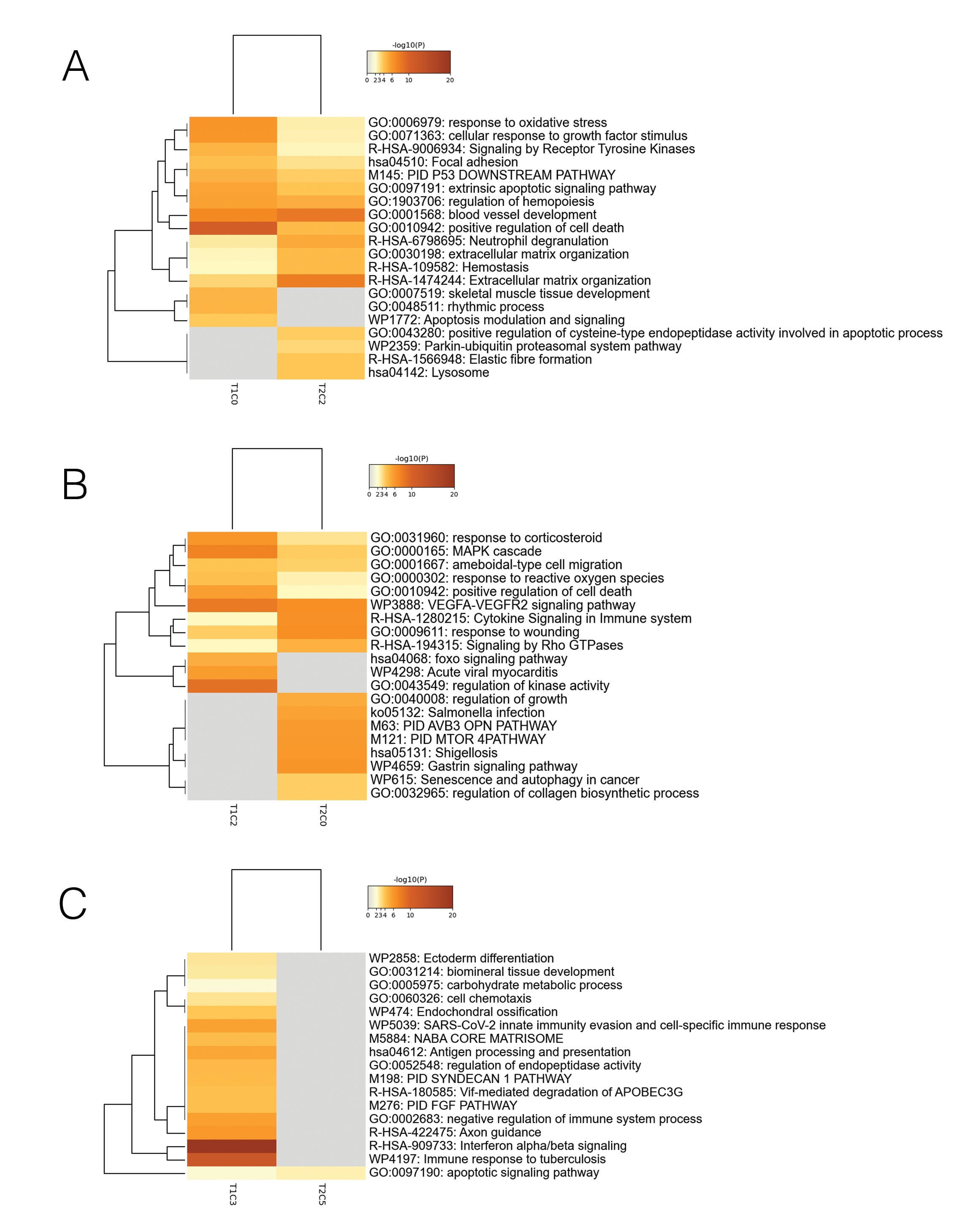

Supplement: Supplementary file 1 [file biology-10-00987-s001.zip › Supplementary_material/supl-fig-6.png]

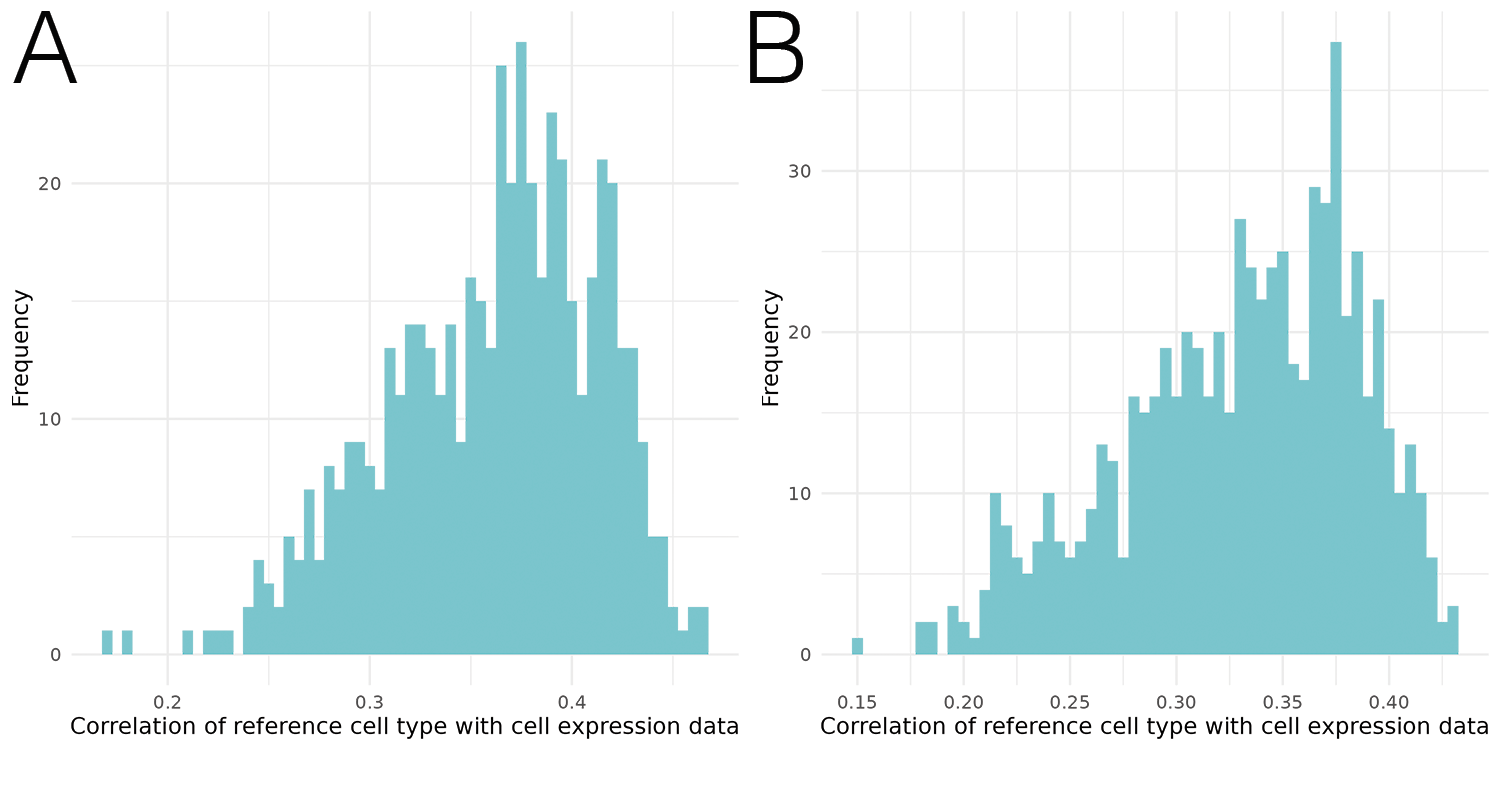

Supplement: Supplementary file 1 [file biology-10-00987-s001.zip › Supplementary_material/supl-fig-7.png]

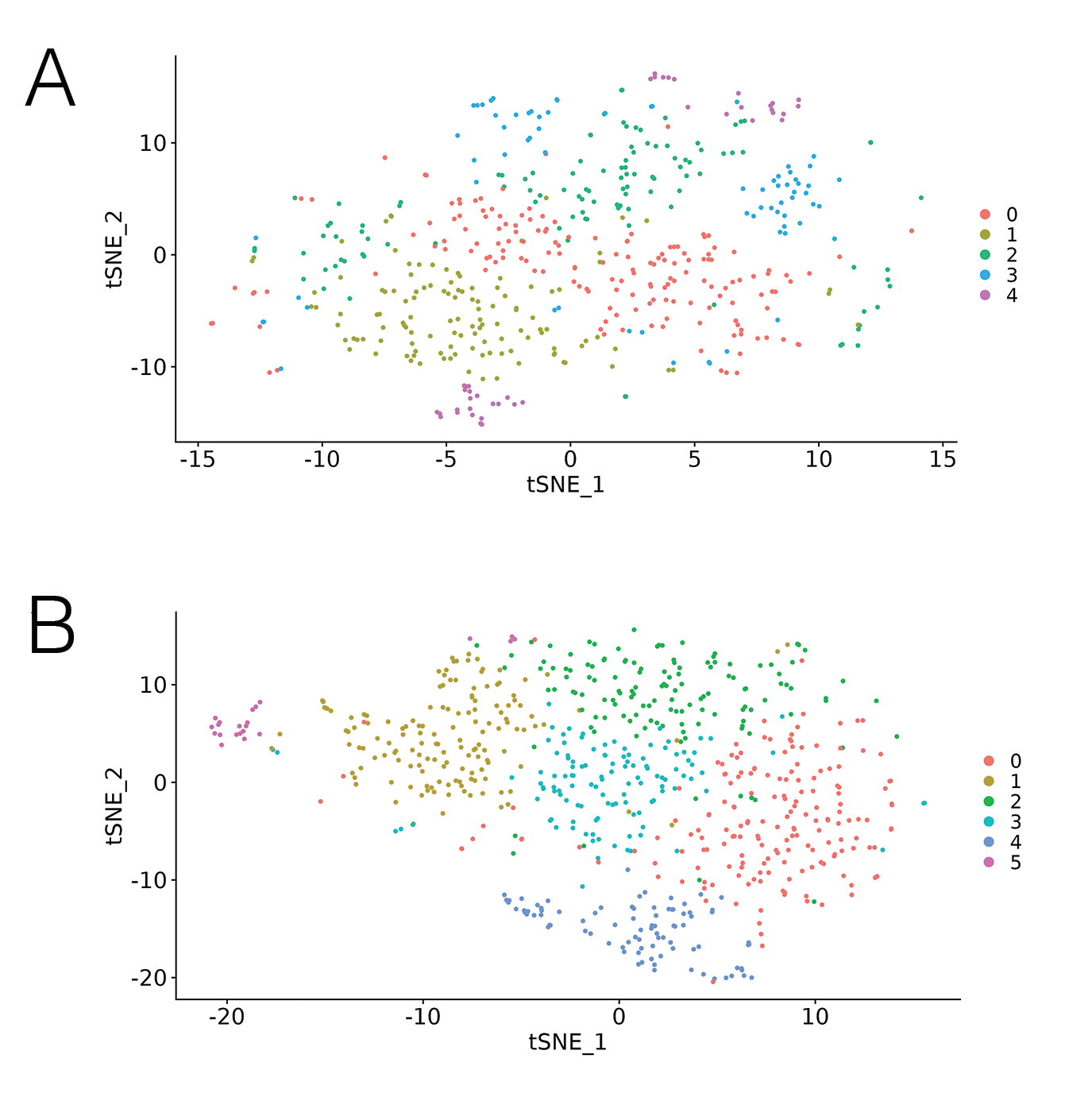

Supplement: Supplementary file 1 [file biology-10-00987-s001.zip › Supplementary_material/supl-fig-5.png]

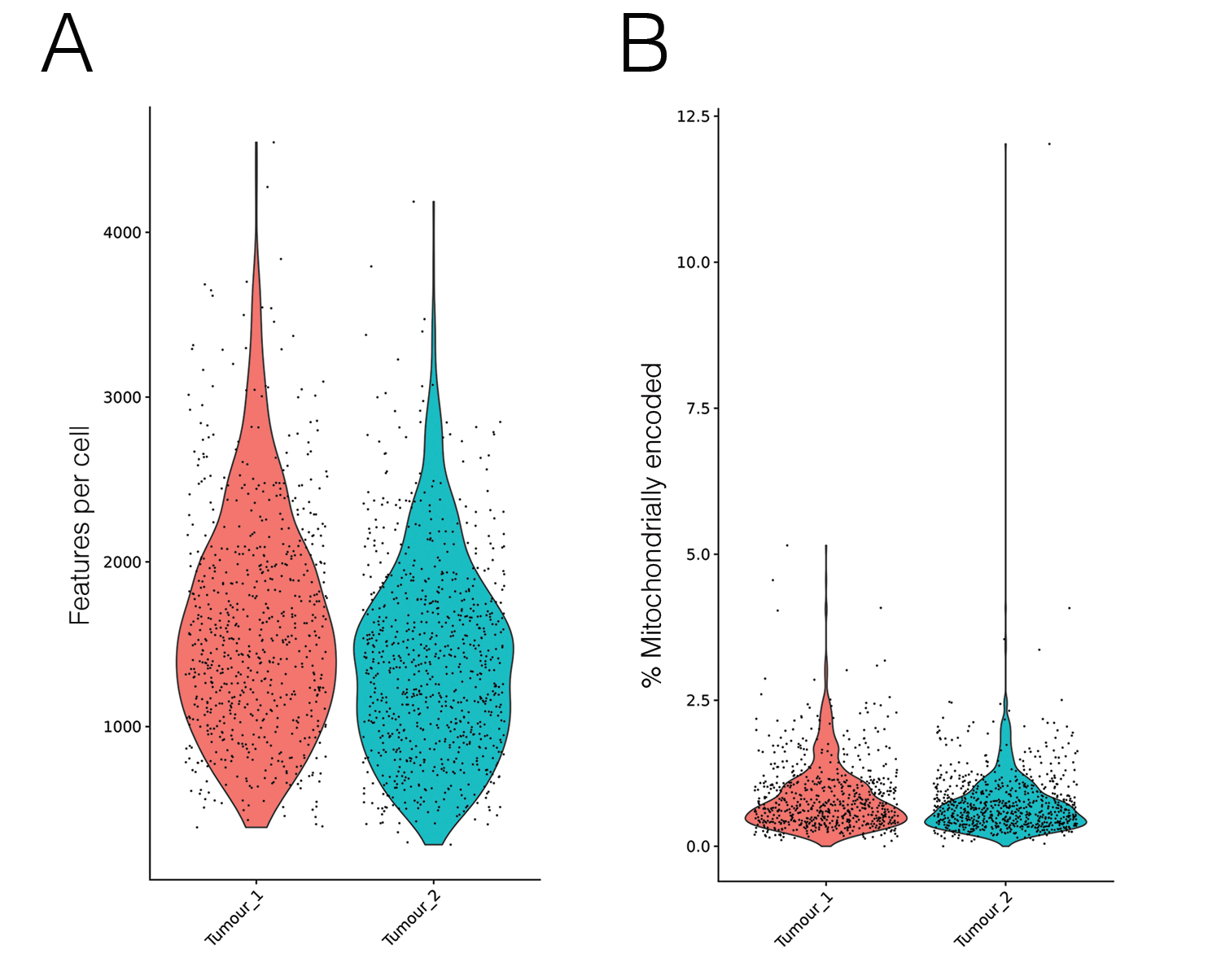

Supplement: Supplementary file 1 [file biology-10-00987-s001.zip › Supplementary_material/supl-fig-4.png]
